# Medical Society of the State of Georgia

**Published:** 1858-05

**Authors:** 


### Medical Society of the State of Georgia.

The Society assembled at 11 o'clock in the town hall, in the town of Madison; was called to order by the 1st Vice-President, Dr. H. F. Campbell of Augusta.

The following regular members present: W. T. Hollingsworth, W. S. Meiere, Jos. P. Logan, A. Means, J. G. Westmoreland, M. H. Oliver, John W. Jones, T. C. H. Wilson, Thomas S. Powell, V. H. Taliaferro, G. L. McClesky, Henry F. Campbell, Eben Hillyer, J. N. Simmons, Juriah Harris, E. E. Jones, D.W. Ford, S. G. Anderson, Wm. L. Jones, J.T. Banks.

The minutes of the last annual meeting held in Augusta, April 8th, 1857, were read and approved. The rules were suspended, and, on written application, the following gentlemen were duly elected members of the society: Drs. R. T. Pulliam, A. J. Shaffer, J. N. Coe, L. I. Green, A. M. Parker, Isham A. Ragan, I. H. Conoly, F. S. Colley, S. J. Saffold, L.

T. P. Harwell, L. G. Anderson, J. M. Boring, S. H. Dean, R. J. Stewart, S. H. Smith, L. S. Means, A. M. Boyd, W. F. Westmoreland, H. W. Brown, B. M. Smith, A. S. Walker, Wm. H. Philpot, Wm. B. Crawford, Gasby Knight.

The election of officers being next in order, the ballots were ordered, and the following gentlemen were elected for the ensuing year: Dr. Jos. P. Logan, of Atlanta, President; Dr. H. J. Ogleby, of Madison, 1st Vice-President; Dr. John T. Banks, of Zebulon, Pike county, 2d Vice-President; Treasurer, Recording and Corresponding Secretary, Dr. Eben Hillyer, of Rome.

On motion, a committee of two were appointed to conduct the President elect, Dr. Jos. P. Logan to the chair. Which being done, he returned his thanks to the society in a few appropriate remarks for the honor conferred upon him.

Dr. E. H. W. Hunter, of Louisville, was, by ballot, elected Orator, and Dr. G. L. McClesky, Alternate, for the next annual meeting.

The selection of delegates to the American Medical Association being next in order, a committee of five—consisting of the following gentlemen: Drs. Ford, Harris, Westmoreland, Simmons and Boyd—were appointed by the Chair to nominate them and report at their earliest convenience.

Society then adjourned until 2 o'clock P. M.

*Afternoon Session.*—Secretary called to order by the President.

Dr. Hillyer, by appointment, read an Essay upon the Physiology of Menstruation.

The Committee appointed to nominate delegates to attend the next meeting of the American Medical Association, to be held in May next, at Washington City, reported the names of the following gentlemen as delegates: Dr. J. Harris, of Savannah; W. T. Hollingsworth, of Morgan; F. S. Colley, of Walton; W. G. Bulloch, of Savannah; H. F. Campbell, of Augusta; A. M. Boyd, of Cave Spring; Eben Hillyer, of Rome; B. M. Smith, of Atlanta; R. T. Pulliam, of Atlanta; J. M. Simmons, of Griffin; T. S. Powell, of Atlanta; C. B. Nottingham, of Macon; R. Q. Dickerson, of Albany; E. M.

Pendleton, of Sparta; Jas. Green, of Macon; J. T. Banks, of Zebulon.

Dr. Meiere, by appointment, read an interesting paper upon the Use of Alcohol in Typhoid Fever.

Society then adjourned until 7 P. M., to hear the annual address, by Dr. T. S. Powell.

*Evening Session.*—Society called to order by the President.

Dr. W. F. Westmorland moved that the mode of appointing Essayists be changed. That they be allowed to write upon upon any subject of their own selection. Which motion was carried.

Upon motion, Drs. Campbell, E. Jones, Oliver, W. F. Westmoreland and Dean were appointed by the Chair a committee to appoint Essayists for the next meeting.

The report of the late treasurer, Dr. Nottingham, was received and adopted.

A ballot was ordered to determine upon a place to hold the next annual meeting, which resulted in the election of Atlanta.

Dr. J. G. Westmoreland moved that the society have published, in pamphlet form, two hundred copies of the constitution, by-laws, code of ethics, etc., of the society.

Dr. Meiere offered as a substitute, that the medical journals at Savannah, Augusta and Atlanta be requested to publish in their columns the constitution, by-laws, etc., of the society, which was adopted.

The Committee on Essays reported the names of the following gentlemen to contribute essays upon any subject of their own selection to the society at the next annual meeting: Drs. H. W. D. Ford, H. F. Campbell, Dr. Smith of Griffin, E. Hillyer, Dr. Stewart of Pike county, G. B. Knight, S. H. Dean, W. F. Westmoreland, E. W. Doughty, J. Harris, J. G. Howard, R. D. Arnold, V. H. Taliaferro, Jos. A. Eve, A. M. Boyd, Jos. P. Logan, H. W. Brown, J. M. Green, T. B. Ford, G. L. McCleskey.

On motion of Dr. Means, it was requested that any gentleman who desired to report the history of any interesting case, or other communication, would do so, and the society would be glad to receive it.

The following communication was received from a Committee appointed by the Faculty of the Savannah Medical College, to ask the co-operation of the society, in having the laws forbidding dissections, repealed or modified, and a law passed legalizing dissections:

*To the President and Members of the*
*Medical Society of the State of Georgia:*

Gentlemen: We, the undersigned committee, have been appointed by the Faculty of the Savannah Medical College, and instructed to confer with you, asking your concurrence in a movement having for its object the passage of a law, by the legislature of Georgia, legalizing dissection for the purposes of medical and surgical study.

To effect this great object, we would most respectfully solicit the society to unite with us in a petition to the legislature at their next session to pass an act rendering it lawful for the professors and teachers in medical colleges and schools in this State, to receive, for anatomical study, the unclaimed bodies of persons dying at public hospitals and other kindred institutions. Such a law is in force in other States.

Accompanying this will be found a copy of the statute enacted by the legislative assembly of the State of New York.

The New York law concedes, as you observe, very little else than the single point of legalizing dissection; even this may be of immense practical service by ridding us of the annoyance to which we may at any time be exposed, whilst the possession of a dead body, even for scientific purposes, is, according to the existing laws, equivalent to a crime to which evil disposed persons might at any time call public attention.

Is it not the bounden duty of the State to see that the medical profession of the land, who are the true missionaries to suffering humanity, lack nothing needful for their efficiency and usefulness, without the necessity of going out of the State to obtain this knowledge, not forgetting that it is the profession alone that can be brought to bear on the waste of human life?

The public demands that the Medical Institutions shall furnish it with good and accomplished surgeons, yet it has set its face against the only means of obtaining them. But, at the present time, under its present prosperity and attainment—there is that advancement made in other branches of learning and science—there is that enlightened policy in legislation upon all matters of general interest and improvement, that we *have a right* now to expect, and to ask for some measure, some means, whereby we can procure material for dissection in some legitimate manner, that we may be enabled to make that progress and advancement in our profession, that will raise it to that rank and standard it so nobly bears in other sections of our land—we wish to be relieved from this false and dangerous position, that affixes upon us the stigma of felons in the acquisition of anatomical knowledge, and all these difficulties and excessive annoyances under which we are laboring, and which we are now obliged to encounter and overcome to make that progress and advancement in our profession, that operates only to favor our more faithful discharge of duty.

This is not for the exclusive advantage of the medical man. It seeks no other benefit than that it wishes all others to enjoy. It is for the common good. Every intelligent man, whether professional or non-professional, cannot but admit that the interest of society imperatively demands the study of practical anatomy. Then, why should not the State make suitable arrangement by law.

Is there any reason why we should stand in this matter behind New York, Massachusetts, Michigan, and many other States in an enlightened policy, considering the rights of the medical profession in the just and proper estimate of its value, and in comparison with the privileges that are extended to it in other States.

Let there be removed from our statute books those laws repressing the study of science—laws that should exist only in darker ages, that now put a barrier in the way, almost ruinous to the pursuit of anatomical investigation, and continually threatens and exposes us to a most disgraceful and ignominious punishment. We would prefer securing the passage of a more liberal law than that of the New York act, so that if our views on the subject accord, any petition your honorable body may think proper to make, calculated to facilitate the passage of a bill through the legislature, would meet our hearty concurrence.

WM. G. BULLOCH, M. D.,
R. D. ARNOLD, M. D.

On motion of Dr. Harris, the professors of anatomy in each one of the Medical Colleges in the State were appointed a committee to confer together, and adopt the best means to carry out the object proposed in the above communication, and also desiring them to petition the legislature upon the subject.

Dr. Taliaferro offered the following, which passed:

*Resolved*, That the thanks of the Society be tendered Col. C. R. Hanleiter, of Atlanta, Editor of the National American, for his presence on this occasion, and for the interest he has manifested in the elevation of medical science, by the exclusion from the columns of his paper, of the advertisements of all quack medicines, secret remedies, criminal drugs, &c., which too frequently pollute the public press.

The following, by Dr. Dean, was passed:

*Resolved*, That the thanks of this Society be tendered to the town authorities of Madison, for the use of their hall; to the trustees of the Presbyterian Church for the use of their church building; to the physicians and citizens for their courtesy and attention to the members of this Society.

Dr. Harris read the following communication from Dr. Arnold, of Savannah:

"SAVANNAH, April 11th, 1858.

*To the Officers and Members of the*
*Medical Society of the State of Georgia:*

GENTLMEN—My engagements with the Savannah Journal have prevented me from fulfilling the appointment of the Society, in writing an article on the Pathology and Treatment of Yellow Fever. I think I can do the subject more justice in carrying out the plan I have formed, of giving in successive numbers of that Journal a full history of our terrible epidemic of 1854, for which my notes taken at the time are ample. I respectfully request to be discharged from the further consideration of the subject.

Respectfully Yours, &c.,

R. D. ARNOLD."

The request was granted.

On motion, the following was passed.

*Resolved*, That this Society do now adjourn, to reassemble in the City of Atlanta, on the 2d Wednesday in April, 1859.

EBEN HILLYER, M. D., *Secretary.*

